# The FEED1 trial: protocol for a randomised controlled trial of full milk feeds versus intravenous fluids with gradual feeding for preterm infants (30–33 weeks gestational age)

**DOI:** 10.1186/s13063-021-05994-z

**Published:** 2022-01-20

**Authors:** Eleanor J. Mitchell, Garry Meakin, Josie Anderson, Jon Dorling, Chris Gale, Rachel Haines, Charlotte Kenyan, Mark J. Johnson, William McGuire, Hema Mistry, Alan Montgomery, Sam Oddie, Reuben Ogollah, Phoebe Pallotti, Christopher Partlett, Kate F. Walker, Shalini Ojha

**Affiliations:** 1 Nottingham Clinical Trials Unit, Building 42, University Park, University of Nottingham, Nottingham, NG7 2RD UK; 2 Bliss, 4th Floor Maya House, 134-138 Borough High Street, London, SE1 1LB UK; 3 Department of Neonatal Medicine, University Hospital Southampton NHS Foundation Trust and NIHR Biomedical research Centre Southampton, University Hospital Southampton NHS Foundation Trust and University of Southampton, Southampton, SO16 6YD UK; 4 Neonatal Medicine, School of Public Health, Faculty of Medicine, Imperial College London, Chelsea and Westminster Hospital campus, London, SW10 9NH UK; 5 c/o Nottingham Clinical Trials Unit, Building 42, University Park, University of Nottingham, Nottingham, NG7 2RD UK; 6 Centre for Reviews and Dissemination, University of York, A/B Block, Alcuin College, York, YO10 5DD UK; 7 Warwick Clinical Trials Unit, Warwick Medical School, University of Warwick, Coventry, CV4 7AL UK; 8 Department of Neonatal Medicine, Bradford Teaching Hospitals NHS Foundation Trust, Duckworth Lane, Bradford, BD9 6RJ UK; 9 Division of Midwifery, School of Health Sciences, University Park, University of Nottingham, Nottingham, NG7 2RD UK; 10 Population and Applied Health Sciences, School of Medicine University of Nottingham, University Park, Nottingham, NG7 2RD UK; 11 Neonatal Unit, University Hospitals of Derby and Burton NHS Foundation Trust, Uttoxeter Road, Derby, DE22 3DT UK

**Keywords:** Clinical trial, Protocol, Preterm infant, Feeding, Neonatal, Full milk, Enteral feeding

## Abstract

**Background:**

In the UK, approximately 8% of live births are preterm (before 37 weeks gestation), more than 90% of whom are born between 30 and 36 weeks, forming the largest proportion of a neonatal units’ workload. Neonatologists are cautious in initiating full milk feeds for preterm infants due to fears of necrotising enterocolitis (NEC). There is now evidence to dispute this fear. Small studies have shown that feeding preterm infants full milk feeds enterally from birth could result in a shorter length of hospital stay, which is important to parents, clinicians and NHS services without increasing the risk of NEC. This trial aims to investigate whether full milk feeds initiated in the first 24 h after birth reduces the length of hospital stay in comparison to introduction of gradual milk feeding with IV fluids or parenteral nutrition.

**Methods:**

FEED1 is a multi-centre, open, parallel group, randomised, controlled superiority trial of full milk feeds initiated on the day of birth versus gradual milk feeds for infants born at 30^+0^ to 32^+6^ (inclusive) weeks gestation. Recruitment will take place in around 40 UK neonatal units. Mothers will be randomised 1:1 to full milk feeds, starting at 60 ml/kg day, or gradual feeds, as per usual local practice. Mother’s expressed breast milk will always be the first choice of milk, though will likely be supplemented with formula or donor breast milk in the first few days. Feeding data will be collected until full milk feeds are achieved (≥ 140 ml/kg/day for 3 consecutive days). The primary outcome is length of infant hospital stay. Additional data will be collected 6 weeks post-discharge. Follow-up at 2 years (corrected gestational age) is planned. The sample size is 2088 infants to detect a between group difference in length of stay of 2 days. Accounting for multiple births, this requires 1700 women to be recruited. Primary analysis will compare the length of hospital stay between groups, adjusting for minimisation variables and accounting for multiple births.

**Discussion:**

This trial will provide high-quality evidence on feeding practices for preterm infants. Full milk feeds from day of birth could result in infants being discharged sooner.

**Trial registration:**

ISRCTN ISRCTN89654042. Prospectively registered on 23 September 2019: ISRCTN is a primary registry of the WHO ICTRP network, and all items from the WHO Trial Registration dataset are included.

## Background

In preterm infants, early establishment of enteral feeding is associated with reduced sepsis, improved growth [1] and enhanced neurodevelopment [2]. Achieving full milk feeds sooner and improving growth without infection or necrotising enterocolitis (NEC) may help the infant be ready for home earlier, reducing the length of hospital stay. Despite evidence to the contrary [3], clinicians often delay initiating feeds or increment feeds slowly due to fear of NEC. The recently completed Speed of Increasing milk Feeds Trial (SIFT) provides firm evidence from over 2800 infants that faster advancement of milk feeds does not increase the risk of NEC even in the most premature infants [4]. In this large, multi-centre trial comparing fast (30 ml/kg/day) vs. slow (18 ml/kg/day) feed increments in 2804 infants born before 32 weeks gestation, the risks of NEC or death were not increased by the faster increment in feeds. Faster fed infants achieved full feeds quicker and received intravenous (IV) fluids for fewer days. A recent Cochrane review assessing advancement of feed volumes in preterm infants concluded that slow advancement of enteral feed volumes results in several days of delay in establishing full milk feeds and may increase the risk of invasive infection [1] possibly due to increased iatrogenic infections from a longer duration of IV line placement.

These results indicate that there may be benefit from a faster approach to feeding. In infants between 30^+0^ to 32^+6^ weeks this could be achieved by providing their full fluid requirements solely as enteral feeds from day of birth without using IV fluids or parenteral nutrition, i.e. “full milk” feeds from the day of birth. A Cochrane review on early full enteral feeding in preterm infants included 6 randomised controlled trials involving 601 stable infants weighing between 1000 and 1500 g at birth [5]. A meta-analysis of length of hospital stay (4 studies, 398 infants) showed a mean difference of − 2.26 days in infants given early full enteral feeds. There was no difference in incidence of NEC. However, all studies were conducted in India and had a moderate to high risk of bias in several areas when assessed for methodological rigour. One small study included in the review included 46 infants (birth weight 1200–1500 g; mean gestation 31 weeks) who were randomised to receive full milk from one hour after birth or IV fluids and slow feed increments (20 ml/kg/d) [6]. They found that infants randomised to full milk feeding regained birth weight quicker, had improved growth at discharge, shorter duration of hospital stay and fewer cases of sepsis without an increased risk of NEC. Additionally, in a trial of 180 infants, Nangia et al. showed a mean difference of − 3.6 days in time taken to reach full enteral feeds in infants fed early enteral feeds and a reduction of 4.1 days in length of hospital stay [7]. These findings suggest it is potentially safe and may be better to start full milk feeds on the day of birth without increasing the risk of NEC and possibly reducing the risk of sepsis. An observational study also showed that implementing full milk feeds from birth is feasible and acceptable, with the infants receiving full enteral milk feeds having significantly fewer cases of NEC and sepsis, less antibiotics, less parenteral nutrition and a shorter average hospital stay [8]. These studies were all conducted in India where the preterm population, healthcare resources, infrastructure and delivery systems as well as treatments and risk factors are different to that in the UK. There have been no studies in the UK or other similar high resource settings investigating the strategy of feeding preterm infants “full milk” feeds from day of birth.

### Justification for study population

In 2018, in England and Wales, there were 656,925 live births of which 51,864 (7.9%) were preterm (born before 37 completed weeks gestation) [9]. Providing optimal nutrition is a cornerstone of neonatal care and the subject of many recent research studies including SIFT [4], ADEPT [10], NEON [11] and SCAMP [12]. These and other similar studies focus on extremely preterm infants (born before 30 weeks) at highest risk of adverse outcomes (death or NEC). However, more than 90% of preterm infants are born at or after 30 weeks, including the 12% of preterm infants who are eligible for FEED1. More mature preterm infants (≥ 34 weeks) typically do not require special care. Infants born at 30, 31 or 32 weeks comprise over 40% of preterm infants routinely admitted to neonatal units (in 2018 there were 5879 infants in this group in England and Wales) and form the largest proportion of workload for neonatal services. Treatments that reduce length of stay in this group of preterm infants could therefore impact the largest number of infants in neonatal units and their families.

### Justification for comparators

Infants that are fully milk fed need less monitoring and could be moved to lower dependency care. Adequate enteral nutrition could promote weight gain and reduce the risk of infections, potentially making infants ready for home sooner and reducing the length of hospital stay. This would make scarce higher dependency cots for sicker infants more readily available and avoid transferring infants further afield to access resources.

Strategies that aim to safely achieve a shorter hospital stay could improve care for all infants who require neonatal care. Full milk feeds may also reduce the cost of care by decreasing use of parenteral nutrition and IV fluids and reducing iatrogenic infections. Preterm birth is associated with long-term morbidities and large lifetime financial costs, placing strain on NHS finances and social care resources. Full milk feeds may improve nutrition and reduce morbidities such as sepsis, thereby improving neurodevelopmental outcomes and lifelong quality of life for this large group of infants. Such an approach that improves the care of preterm infants whilst simultaneously reducing the cost of care would achieve the NHS “Five Year Forward View” aim of achieving efficiency savings whilst maintaining and improving quality of care and safety [13]. There are also potential benefits for mothers and families from a less medical model of care, with opportunities for involvement in care, shorter mother-infant separation and improved satisfaction and mental health outcomes.

The FEED1 Trial addresses three of the top six research priorities identified by the James Lind Alliance [14]:

- “What is the optimum milk feeding strategy and guidance (including quantity and speed of feeding and use of donor and formula milk) for the best long-term outcomes of premature infants?
- How can infection in preterm infants be better prevented?
- Which interventions are most effective to prevent necrotising enterocolitis in premature infants?”

This paper is reported in accordance with the Standard Protocol Items: Recommendations for Interventional trials (SPIRIT) guidelines [15].

### Objectives

To determine whether, in infants born at 30^+0^ to 32^+6^ weeks gestation, full milk feeds initiated in the first 24 h after birth reduces the length of infant hospital stay in comparison to IV fluids or parenteral nutrition with gradual milk feeding.

## Methods/design

This is a multi-centre, open, parallel group, randomised (1:1) controlled superiority trial of full milk feeds versus gradual milk feeds. Mothers and infants will be recruited from around 40 neonatal units in the UK. A list of participating sites can be found at www.feed1.ac.uk. The full protocol and other trial documentation, including participant information sheets and consent forms, can also be found on the trial website. An embedded Study Within a Trial (SWAT) is also included which is investigating methods of training sites. Details of the SWAT can be found on the SWAT repository [16, 17] and will be reported separately.

### Eligibility

Inclusion criteria include (i) infants born at 30^+0^ to 32^+6^ weeks gestation (inclusive) and ii) infants < 3 h (180 min) old (since recorded time of birth). Infants requiring respiratory support (such as via continuous positive airway pressure) or other supportive treatments will be included in the study if the clinician is in equipoise about the infant being randomised to either the “full milk” or the “gradual milk” arm. Similarly, well infants should only be included if the attending clinician is in equipoise about the best feeding regime and the infant being randomised to either “full milk” or “gradual milk” groups. Exclusion criteria include (i) infants with known congenital abnormalities of the gastrointestinal tract or other congenital conditions that make enteral feeding unsafe, (ii) infants who are small for gestational age (SGA) (birth weight < 10^th^ centile) and have evidence of reversed end-diastolic flow on antenatal umbilical artery Doppler ultrasound and (iii) mothers who have participated in the trial during a previous pregnancy.

### Interventions

For infants in the full milk group (intervention), fluids will be started as milk at 60 ml/kg/day, increased as per their individual requirements and in line with standard neonatal practice. The choice of feeding intervals will be determined by local policy and clinician’s preference. Wherever possible, mother’s expressed breast milk will always be the first preference for infant milk feeds. It is likely that mother’s breast milk will need to be supplemented with additional milk, i.e. either infant formula milk or donor breast milk in the first few days. The decision as to the type of milk used will be made by the mother and the site, in line with the site’s local policy. For infants in the gradual milk group (control), fluids will be given in accordance with standard practice at the site. This may include milk feeds, starting at a maximum of 30 ml/kg/day on day 1 with a minimum of 30 ml/kg/day of supplementary IV fluids or parenteral nutrition. Adherence to the randomised allocation will be monitored on a regular basis by the Trial Management Group. Due to the pragmatic nature of this neonatal feeding trial, there are no prohibited interventions or concomitant medications.

### Outcomes

The primary outcome is length of infant hospital stay. Since hospitals could apply different discharge criteria, a secondary outcome of time until objective discharge criteria are met has also been included. The statistician, blinded to treatment allocation, will use the date at which each infant first met all three of the following criteria: (i) current weight ≥ 1700 g, (ii) infant is able to take at least one full suck feed assessed as adequate and (iii) infant has been off additional temperature support for ≥ 24 h. Daily data will be collected to determine the day on which the infant achieved all the three features to determine the time until objective discharge criteria are met. The primary and all secondary outcomes, including the five elements (domain, specific measurement, specific metric, method of aggregation and time point) as defined by Saldanha et al [18], are listed in Table 1.

**Table 1:** Primary and secondary outcome measures

Domain	Specific measurement	Specific metrics	Method of aggregation	Time point
**Primary outcome**				
Length of hospital stay	Date of hospital discharge	Difference between the date the infant is discharged and date of randomisation	Mean and standard deviation (SD), per group	Hospital discharge
**Secondary outcomes**				
Survival	Date of death as recorded on eCRF. Date of death as recorded on eCRF	Death between date of randomisation and date of hospital discharge. Death between date of randomisation and 6 weeks corrected gestational age	Proportion of infants alive, per group. Proportion of infants alive, per group	Hospital discharge 6 weeks corrected gestational age
Microbiologically confirmed (positive blood/cerebrospinal fluid culture) or clinically suspected late-onset sepsis	Presence of confirmed or suspected late-onset sepsis on microbiology report and recorded in eCRF^1^	Microbiologically confirmed late-onset sepsis between date of randomisation and hospital discharge	Proportion of infants with microbiologically confirmed late-onset sepsis, per group	Hospital discharge
Necrotising enterocolitis (NEC) (Bell’s stage 2 or 3) [19]	Presence of NEC (stage 2 or 3) recorded on eCRF^1^	Diagnosis of NEC (stage 2 or 3) between date of randomisation and hospital discharge	Proportion of infants with NEC (stage 2 or 3), per group	Hospital discharge
Time taken to maintain full milk feeding	Full milk feeds reached, defined as at least 140 ml/kg/day for three consecutive days^2,^ as recorded on eCRF	Derived from the date the infant achieves full feeds, recorded on CRF, and date of randomisation	Mean (and SD) and/or median (and IQR), per group	During hospital admission, in accordance with criteria for reaching full milk feeds
Time to regain birth weight	Birth weight regained, as recorded on eCRF	Derived from the date the infant regains birth weight and date of randomisation	Mean (and SD) and/or median (and IQR), per group	During hospital admission
Growth of infant	Growth *Z* scores (length, weight and head circumference), corrected for gestational age as per UK-NICM growth charts [20]	Derived from growth *Z* scores, expected date of delivery and infant sex	Mean (and SD) and/or median (and IQR), per group	Hospital discharge
Breast-feeding	Infant being breast-fed, as recorded on CRF. Infant being breast-fed, as recorded on 6-week parent-completed questionnaire	Infant being breast-fed at time of hospital discharge. Infant being breast-fed at 6 weeks (corrected gestational age)	Proportion of infants breast-feeding, per group. Proportion of infants breast-feeding, per group	Hospital discharge. 6 weeks corrected gestational age
Breast milk feeds	Infant being fed mother’s breast milk, as recorded on eCRF. Infant being fed mother’s breast milk, as recorded on 6-week parent-completed questionnaire	Infant being fed mother’s breast milk at time of hospital discharge. Infant being breast fed mother’s breast milk at 6 weeks (corrected gestational age)	Proportion of infants being fed mother’s breast milk, per group. Proportion of infants being fed mother’s breast milk, per group	Hospital discharge. 6 weeks, corrected gestational age
Number of days of cannulae	Number of days of peripheral cannula and IV cannulae inserted, as recorded on eCRF	Number of days of peripheral cannula and IV cannulae inserted, until infant reaches full milk feeds	Mean (and SD) and/or median (and IQR), per group	Date infant reaches full milk feeds, whilst in hospital
Number of days of infant receiving parenteral nutrition	Number of days of infant receiving parenteral nutrition, as recorded on eCRF	Number of days of infant receiving parenteral nutrition until hospital discharge	Mean (and SD) and/or median (and IQR), per group	Hospital discharge
Number of central venous lines inserted	Number of central venous lines, including umbilical and percutaneous or surgically inserted venous lines), as recorded on eCRF	Number of central venous lines between date of randomisation and date of hospital discharge	Mean (and SD) and/or median (and IQR), per group	Hospital discharge
Number of central line days	Number of days infant has a central line, as recorded on eCRF	Number of central line days between date of randomisation and date of hospital discharge	Mean (and SD) and/or Median (and IQR), per group	Hospital discharge
Time until objective discharge criteria are met	Objective discharge criteria, as recorded on eCRF	Derived from the date the objective discharge criteria is met and date of randomisation	Mean (and SD) and/or median (and IQR), per group	During hospital admission
Length of neonatal unit stay	Length of stay in (i) neonatal intensive care, (ii) high dependency care, (iii) special care, (iv) translational care, as recorded on eCRF	Derived from days spent in each type of neonatal unit between date of randomisation and date of discharge	Mean (and SD) and/or median (and IQR), per group	Hospital discharge
Retinopathy of prematurity (ROP)	Diagnosis of ROP, as recorded on eCRF	Diagnosis of ROP between date of randomisation and date of hospital discharge	Proportion of infants with ROP, per group	Hospital discharge
Chronic lung disease (CLD)	Diagnosis of CLD, as recorded on eCRF	Diagnosis of CLD between date of randomisation and date of hospital discharge	Proportion of infants who are mechanically ventilated or on nasal CPAP or in supplemental oxygen at 36 weeks corrected gestational age, per group	Hospital discharge
Brain injury	Diagnosis of intraventricular haemorrhage (grade 3 or 4), periventricular leukomalacia or hydrocephalus (requiring a shunt) on cranial ultrasound, recorded on eCRF	Diagnosis of brain injury (as per definitions) between date of randomisation and date of hospital discharge	Proportion of infants with recorded evidence of brain injury (grade 3 or 4 intraventricular haemorrhage, periventricular leukomalacia or hydrocephalous requiring shunt, per group	Hospital discharge
Blood glucose/hypoglycaemia	Number of blood glucose tests, as recorded on eCRF. Number of blood glucose tests indicating hypoglycaemia (< 2.2 mmol/L). Number of blood tests indicating severe hypoglycaemia (< 1.0 mmol/L)	Number of blood glucose tests, between date of randomisation and date of hospital discharge. Number of blood glucose tests < 2.2 mmol/L, between date of randomisation and date of hospital discharge. Number of blood glucose tests < 1.0 mmol/L between date of randomisation and date of hospital discharge	Mean (and SD) and/or median (and IQR), per group. Mean (and SD) and/or median (and IQR), per group. Mean (and SD) and/or median (and IQR), per group	Hospital discharge. Hospital discharge. Hospital discharge
Hospital visits	Number of hospital visits, including day care and overnight admissions, as recorded on 6-week parent-completed questionnaire	Number of hospital visits between date of hospital discharge and 6 weeks corrected gestational age	Mean (and SD) and/or median (and IQR), per group	6 weeks corrected gestational age
Parental satisfaction and wellbeing	Preterm birth experience and satisfaction scale (P-BESS) [21], completed by parents	P-BESS total score (and subscales for interpersonal care, information and explanations, lack of confidence in staff) at 6 weeks corrected gestational age	Mean (and SD) and/or median (and IQR), per group	6 weeks corrected gestational age

^1^Indeterminate cases will be subject to a blinded endpoint review to make a final determination

^2^Infants who are partially or exclusively breast fed will be considered to have achieved full enteral milk feeds if their intake of milk (breast feeding plus measured volume of additional milk) is considered equivalent to full enteral milk feeds

Outcome measures included have been guided by the Core Outcomes in Neonatology (COIN) core outcome set [22]. Discussions are ongoing to obtain separate funding to collect longer-term follow-up data at 2 years corrected gestational age. In addition, the research team are in discussions with researchers based in other countries, such as Canada and Australia, to set-up parallel studies. This will be to provide sufficient power to assess outcomes of NEC and sepsis; these outcomes occurred in 1% and 12% of infants retrospectively in the SIFT Trial among those who would have been eligible for FEED1.

### Sample size and recruitment

Our sample size calculation has been based upon detecting a between group difference in means of length of stay of 2 days. Input from our parent representatives suggests that from a family perspective even a short reduction in length of hospital stay could make a huge difference to parents. In addition, from a cost-saving perspective, a reduction in length of stay of 2 days for this population of infants could result in £5.6 m annual savings for the NHS in England and Wales and > 12,000 days of increased neonatal cot capacity.

Data from audits and previous studies suggest that the distribution of length of hospital stay in this population is approximately normal. To detect a difference in means of 2 days between the two groups with 90% power, 1:1 allocation and 5% two-sided significance requires 1778 infants, assuming a standard deviation of 13 [23, 24]. Allowing for 2% non-collection of the primary outcome data due to death, no consent for data collection after oral assent and infants remaining in hospital at the end of data collection and accounting for clustering will require 2088 infants and recruitment of approximately 1770 women. The inflation to account for clustering assumes that 15% and 1.4% of pregnancies will be twin and triplets respectively and that the intracluster correlation coefficient for length of hospital stay for infants from the same pregnancy is 0.82 [4].

### Participant enrolment and consent

**Fig. 1:**
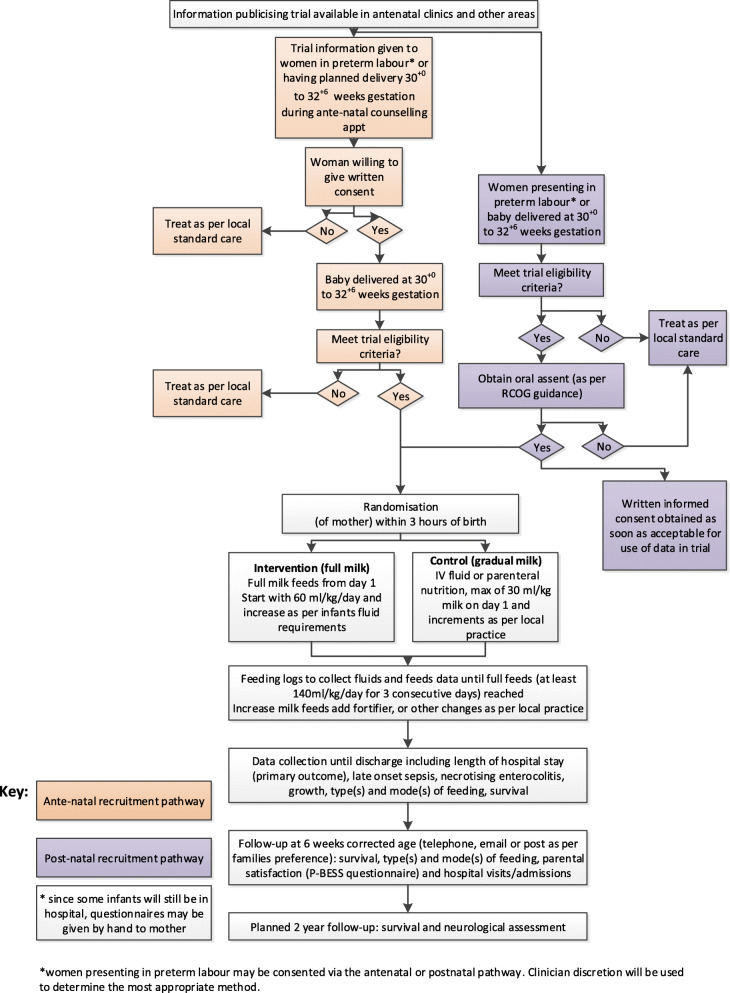
Participant flow diagram

The flow of women and infants throughout the trial is shown in Fig. 1. As the infant must be randomised within three hours of birth, a time during which the woman is recovering from giving birth and that is emotionally fraught and potentially difficult for families, a two-stage consent pathway will be used. Wherever possible, women will be approached antenatally and asked to consider participation in the trial. At around the time of antenatal counselling consultation, women will be given study information, have the opportunity to discuss the study and ask questions and give full written informed consent if they are willing to do so. For these women, once they have given birth, infant eligibility will be checked and the infant(s) will be entered into the trial, unless the mother expresses that she has changed her mind. For some women, receiving information and providing consent antenatally may not be possible due to the rapid and unexpected nature of preterm birth. During labour and postnatally, these women can be approached via an oral assent pathway. This involves a member of the neonatal team inviting the woman to participate and giving the minimal information required to make a decision. A shorter, simplified participant information sheet and/or a short animation film may be used to support this discussion. The decision to participate will be documented in the medical notes and full written informed consent will be obtained later (ideally ≤ 72 h). This two-stage consent pathway has been used successfully in a previous neonatal trial [24] and features as part of guidance from the Royal College of Obstetricians and Gynaecologists (RCOG) [25]. Sites will be fully trained in the two-stage consent pathway and will be provided with a range of supplementary materials to support oral assent conversations which include a recorded webinar, video showing examples of discussions and additional documentation. Women are asked to give their optional consent to be contacted for longer-term follow-up of their infant(s) and for later educational outcomes.

### Randomisation and blinding

The unit of randomisation is the mother to ensure that a mother-infant(s) dyad is treated as a unit. This will also enable antenatal consent and facilitate early commencement of the intervention. In addition, it will ensure that siblings from multiple pregnancies are assigned to the same group. Parents have told us this is important to them as they would not like to feed their infants differently unless there was a medical reason to do so. Randomisation will be performed on a 1:1 ratio, using a secure web-based system, developed and maintained by the Nottingham Clinical Trials Unit (NCTU), which will conceal allocation sequence. Randomisation will use a minimisation algorithm, with a random element, to ensure balance on important prognostic factors: neonatal unit, single or multiple birth, gestational age at birth, birth weight centile and whether IV fluids were started prior to randomisation. Randomisation will be undertaken by the principal investigator, clinician or other study team member within three hours from the recorded time of birth. This is to ensure infants randomised to full milk feeds can receive the intervention with minimal risk of receiving IV fluids and should help prevent contamination between groups.

It is not possible to blind investigators and families due to the nature of the intervention. Unblinding is therefore not relevant as this is an open-label trial. However, the trial statistician will remain blinded throughout the trial. To objectively assess the primary outcome, a secondary outcome measure to objectively assess discharge criteria is included and a blinded endpoint review committee (BERC) will also be established to examine cases of late-onset sepsis and necrotising enterocolitis where the diagnosis is unclear.

### Trial assessments and procedures

All trial assessments and procedures are outlined in Table 2. Most outcome data will be collected during the infant’s hospital admission. A daily feeding log will be completed until the infant receives at least 140 ml/kg/day of feeds, sustained for 3 consecutive days. For infants who are transferred to another hospital, i.e. a continuing care site, a paper transfer pack will accompany the infant with information on trial participation and data collection will continue. To collect data at 6 weeks corrected gestational age, an online questionnaire (or paper if preferred) will be sent to women; reminders will be sent to increase response-rates.

**Table 2:**
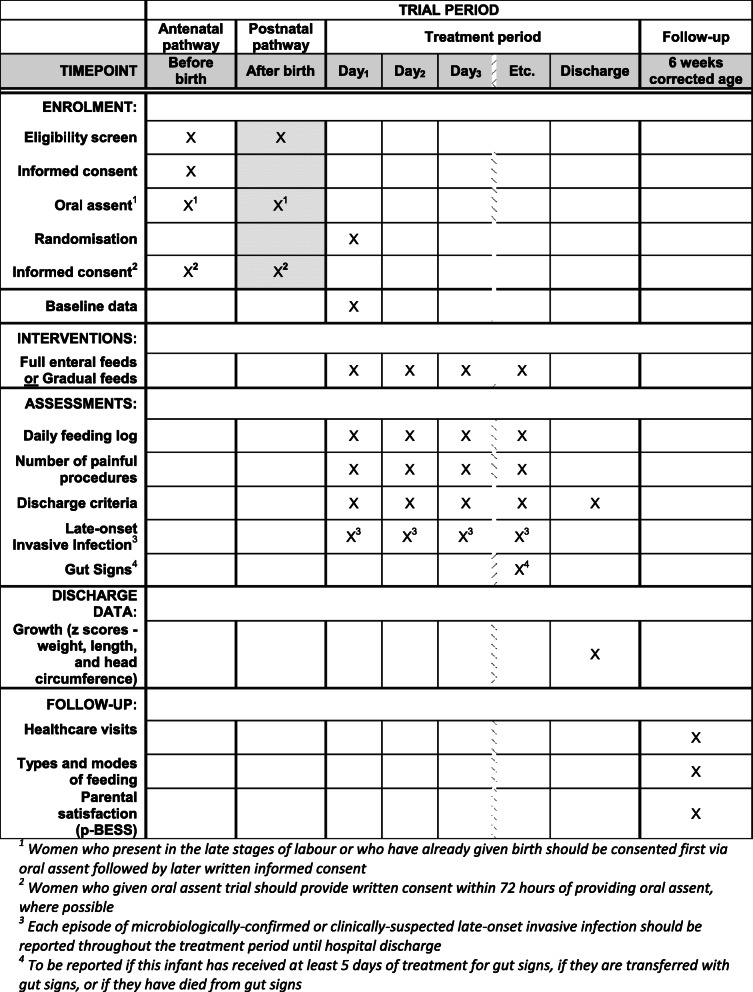
Schedule of assessments

No laboratory specimens will be collected specifically for the trial. Any samples taken are in line with usual care and results will only be recorded in the eCRF if relevant (e.g. blood glucose, late-onset sepsis).

No specific provision has been included for the care of infants outside of the trial; clinical care will be as per usual care, in line with the pragmatic nature of the trial.

### Adverse events

As adverse events are commonly encountered in preterm infants receiving neonatal care, they will be recorded in the infant’s medical notes as per usual practice, from the commencement of the randomised feeding strategy until hospital discharge. Adverse reactions are thus collected as outcomes and will not need to be reported separately via an adverse event reporting process. Blood glucose levels will be monitored routinely for all infants, recorded in the trial eCRF and provided in meeting reports to the independent data monitoring committee (DMC). Coding of adverse events (e.g. MedDRA) will not be undertaken. Serious adverse events (SAEs), including death, will be reported as such by the principal investigator, or delegate, reporting within 24 h of being made aware of the event, using an SAE form, sent via email to a dedicated SAE reporting mailbox at NCTU. Reporting will be aligned with the standard operating procedure (SOP) at the Nottingham Clinical Trials Unit. The chief investigator, or delegate (neonatologist), has responsibility for the review of all SAEs, to assess causality. Late-onset sepsis (microbiologically confirmed or clinically suspected), necrotising enterocolitis (Bell’s stage 2 or 3) or other known complications of prematurity will not be reported as serious adverse events. Only SAEs that are deemed to be related to the trial interventions will be followed up until resolution. If an SAE is unexpected, it will be classified as a SUSAR (serious unexpected suspected adverse reaction). All SUSARs will be reported by NCTU to the ethics committee within 15 days of being notified, and all principal investigators will be notified. The DMC will review all reported SAEs and neonatal outcome data at regular intervals throughout the trial, in line with the DMC charter.

Insurance and indemnity for infants and NHS trial staff is covered within the NHS Indemnity arrangements for clinical negligence claims in the NHS. No special compensation arrangements apply to this trial, though parents may have recourse to the NHS complaints procedure.

### Data management

All trial data will be collected by site staff delegated to do so and entered onto a trial specific database (eCRF) with infants only identified by their unique trial number and initials. Using MACRO (Elsevier), the database will be developed and maintained by NCTU staff. Access to the database will be restricted and secure. Sites will be provided with paper workbooks to assist them with data collection. Missing or spurious data will be queried in a timely manner throughout the trial, in accordance with the trial data management plan. To facilitate contact with families at 6 weeks corrected gestational age, contact details will be collected and entered into an online secure system, developed and maintained by NCTU staff. This data is held separately to the deidentified trial data collected. Access to contact details is restricted to those involved in the follow-up phase, as authorised by the chief investigator.

### Statistical analysis

Analysis and reporting of the trial will be in accordance with the Consolidated Standards of Reporting Trials (CONSORT) [26] guidelines. All analyses will be outlined in a detailed Statistical Analysis Plan agreed prior to database lock. No interim analyses are planned. The primary comparative analyses will be conducted according to randomised allocation with due emphasis on confidence intervals for between-group comparisons.

The primary outcome will be analysed using linear mixed models to compare the mean length of hospital stay between groups, adjusting for minimisation variables and accounting for the correlation between outcomes for infants born from a multiple pregnancy. The estimated between group effect will be presented using the difference in means, with a 95% confidence interval. Secondary outcomes will be analysed similarly using appropriate multilevel regression models, dependent on the type of outcome variable. The between group effect will be reported using an appropriate effect estimate along with a corresponding 95% confidence interval.

The primary approach to between-group comparative analyses will be by modified intention-to-treat (i.e. including all participants who have been randomised and without imputation of missing outcome data). In particular, the primary analysis will exclude the small number of deaths that might occur before discharge, but sensitivity analysis will be performed by imputing the primary outcome with the worst observed length of stay for infants who died prior to discharge to check that this does not influence the findings. A further sensitivity analysis will assess the effect of compliance with the allocated feeding strategy through complier average causal effect (CACE) analysis.

Appropriate interaction terms will be included in the primary regression analyses to conduct subgroup analyses according to gestation at birth and birth weight centile, but this analysis will be regarded as exploratory as the study is not powered to detect interactions. Interpretation of any subgroup effects will be based on the treatment-subgroup interaction and 95% confidence interval.

A within-trial economic analysis will be conducted from an NHS and personal social services perspective. Resource data will be collected prospectively and unit costs will be obtained from routine sources. The main cost-effectiveness analysis will be based on the cost per reduction in days in care. A longer-term projection of costs and benefits will be estimated through decision analytical modelling. Appropriate sensitivity analyses will be undertaken to account for any uncertainty.

### Monitoring and governance

An independent DMC will review unblinded trial data, including safety data, on an intermittent basis. The role of the DMC is outlined in a charter, available upon request, which outlines their terms of reference. Overall independent oversight will be provided by a trial steering committee (TSC).

On-site monitoring will not be conducted routinely throughout the trial. Instead, regular central monitoring of trial data will be undertaken and this data will be used to assess if sites have met any triggers to activate a triggered monitoring visit. The Trial Management Group (TMG) are responsible for reviewing central monitoring reports and agreeing if triggered on-site monitoring visits are required. Any trial conduct audits will be carried out by the sponsor as per their local auditing plans.

### Protocol amendments

Any amendments to the protocol will be managed in line with standard operating procedures at the NCTU. A decision to make an amendment to the protocol will be taken by the Trial Management Group, and changes to the protocol and/or trial documentation will reviewed by the trial sponsor and funder. It is the responsibility of the trial manager, or delegate, to submit the protocol and/or trial documentation for review by the ethics committee and to participating sites. Once an amendment is approved, all documentation relating to the amendment will be sent to Principal Investigators. The trial registry will remain up-to-date throughout the trial, by the trial manager or delegate.

### Patient and public involvement (PPI)

Our research team includes a parent who has experience of having a preterm infant, born at 31 weeks gestation, and the research manager for Bliss, the UK’s largest premature and sick baby charity, who represent parents with experience of preterm birth. In addition to our immediate research team PPI members, we have a group of three parents who provide additional parent input, and a PPI member on the independent trial steering committee. We involved parents throughout the design stage of this trial and continue to do so whilst it is being conducted.

### Dissemination

The results of the FEED1 trial will be shared widely. Participants will receive a lay newsletter, with input from PPI members, unless they have expressed they do not wish to receive this. The results will be made publicly available via the trial website. Participating sites will receive a summary of the results. The trial results will be submitted to a high-impact peer-reviewed scientific journal and to international and national neonatal and paediatric conferences. Results will also be disseminated via social media platforms, including the NCTU Twitter page, FEED1 Trial Twitter page and partner organisations including Bliss. Any requests for data sharing of trial data will be handled in line with the standard operating procedure at the NCTU.

## Discussion

This is a pragmatic trial that has had strong patient and public involvement input from the outset. Having a preterm infant is a stressful, emotional experience and it has therefore been crucial for us to ensure the views of preterm infants are represented and that parents find the trial acceptable. The results of this trial will provide high-quality evidence on feeding practices for preterm infants of 30–33 weeks gestation. Infants in the intervention group may be discharged from hospital sooner and parents have told us that even a small reduction in the length of hospital stay is important to them. In addition, a reduction in length of stay could result in increased cot capacity on neonatal units and a potential cost saving of £5.6.8 million annually (based upon a reduction of 2 days). Full milk feeds from birth may be an intervention that improves outcomes and care of preterm infants and their families whilst simultaneously reducing the cost of NHS care.

Since trial conception one challenge has been with respect to the type of milk available to the infant, since the intervention involves feeding infants full volumes of milk from day of birth. As in standard practice, the first choice will always be the mother’s own milk unless there are medical contraindications or the mother chooses to feed her infant with formula milk. It is likely for some infants that mother’s own milk may not be sufficient in volume during the first few days of life. For infants in the intervention arm, where this is the case, formula milk or donor breast milk will be used to replace the intravenous fluids or parenteral nutrition. Mothers will be supported in breast feeding and full milk feeding with mother’s expressed breast milk will be established as soon as sufficient volumes are available. Breast feeding will be supported and encouraged in both arms of the trial. Information is included about the benefits of breast feeding in all study information. There is no involvement of any kind by any formula milk manufacturers or related organisations in this trial. Several outcomes related to breastfeeding are included. In addition, we will also undertake additional exploratory analyses to compare the primary outcome and key secondary outcomes between infants who have received donor breast milk and preterm formula milk to supplement mother’s breast milk.

### Trial status

Protocol version 1.3, dated 14 May 2020. Recruitment opened on 15 October 2019 and is expected to continue until September 2022 at the earliest. Recruitment paused on 20 March due to the COVID19 pandemic and re-started on 3 July 2020.

## Data Availability

Data sharing is not applicable to this article as no datasets were generated or analysed during the current study. Anonymised participant data will be made available, upon request, in accordance with the NCTU standard operating procedure after publication of trial results.

## Abbreviations

BERC: Blinded endpoint review committee
CLD: Chronic lung disease
DMC: Data monitoring committee
eCRF: Electronic case report form
IQR: Interquartile range
IV: Intravenous
LOS: Late-onset sepsis
NCTU: Nottingham Clinical Trials Unit
NEC: Necrotising enterocolitis
NHS: National Health Service
ROP: Retinopathy of prematurity
SD: Standard deviation
SWAT: Study Within a Trial
TMG: Trial Management Group
TSC: Trial steering committee
UK-NICM: United Kingdom Neonatal and Infant Close Monitoring
